# Resiliency of fast-growing and slow-growing genotypes of broiler chickens submitted to different environmental temperatures: growth performance and meat quality

**DOI:** 10.1016/j.psj.2023.103158

**Published:** 2023-10-06

**Authors:** A. Huerta, A. Pascual, F. Bordignon, A. Trocino, G. Xiccato, A. Cartoni Mancinelli, C. Mugnai, F. Pirrone, M. Birolo

**Affiliations:** ⁎ Department of Agronomy, Food, Natural Resources, Animals and Environment (DAFNAE), University of Padova, 35020 Legnaro, Padova, Italy; † Department of Comparative Biomedicine and Food Science (BCA), University of Padova, 35020 Legnaro, Padova, Italy; ‡ Department of Agricultural, Environmental, and Food Science, University of Perugia, 06121 Perugia, Italy; § Department of Veterinary Sciences, University of Turin, 10095 Grugliasco, Torino, Italy

**Keywords:** local breed, sex, heat stress, myopathy, sensory analysis

## Abstract

Growth performance and meat quality were assessed in 238 chicks of both sexes belonging to a commercial crossbreed (Ross 308), and 2 Italian local breeds (Bionda Piemontese—**BP** and Robusta Maculata—**RM**). The chickens were kept in 2 rooms at standard environmental conditions or under heat stress (+4.7°C on average) until slaughtering (42 d of age for Ross 308 and 99 d for RM and BP chickens). The Ross chickens showed the highest final live weight, feed intake, and daily weight gain, and the best feed conversion ratio compared to the local breeds (*P* < 0.001), with RM performing better than BP chickens. Thus, Ross chickens had the heaviest carcasses, the highest slaughter, and breast yields followed by RM and BP chickens (*P* < 0.001). At the *pectoralis major* (***p. major***) muscle, Ross chickens showed the highest pH, lightness, and yellowness, besides the highest cooking losses, whereas BP showed the highest redness (*P* < 0.001). Ross meat had higher water and ether extract contents, and lower crude protein content compared to BP and RM (*P* < 0.001), whereas no differences among genotypes were measured for the fatty acid profile. At the sensory analysis, Ross breasts had a higher juiciness compared to BP ones, besides a lower score for “brothy and chickeny/meaty” and a higher one for “wet feathers” compared to local breeds (0.05 < *P* < 0.001). The increase of the room temperature decreased growth performance and cold carcass weight (*P* < 0.001) compared to standard conditions, whereas the rate of α-linolenic acid in the meat increased (*P* < 0.01). The effect of a high environmental temperature on growth performance and slaughter and meat quality traits was more pronounced in Ross compared to BP and RM chickens (0.05 < *P* <0.001; significant interaction genotype × temperature). In conclusions, local chicken breeds showed lower performance and slaughter yield compared to the commercial genotype, but more favorable meat quality traits and higher resilience to the environmental heat-stress.

## INTRODUCTION

Global outlooks forecast a constant raise of the demand for meat proteins, due to an increase of population and incomes, changes in home habits, and needs for food security (FAO, 2022). Poultry meat will cover large part of this demand, due to its competitive production and costs, absence of cultural or religious obstacles to consumption, healthy nutritional profile (high protein, low fat and cholesterol contents, and balanced n-3/n-6 fatty acid ratio), and sensorial properties (texture, color, and flavor) (Petracci et al., 2013). In the last 50 years, genetic selection of broiler chickens has been based on fast growth rates and feed efficiency, coupled with high carcass and breast yields (Tixier-Boichard, 2020). However, these selected genotypes are highly susceptible to stress; they decrease their production under less than optimal environmental conditions (Mikulski et al., 2011; Soleimani et al., 2011). The stress responses are associated with some welfare and health issues, impaired walking ability, cardiovascular problems, ineffective thermoregulation, and occurrence of muscle abnormalities (Petracci et al., 2015; Hartcher and Lum, 2020).

The current challenging environmental and geopolitical scenarios are calling for sustainable productions based on animals resilient to climate changes, with special emphasis on heat stress, and on alternative feeds, other than conventional raw materials. Slow-growing chicken genotypes are expected to adapt to challenging environmental and nutritional conditions (Castellini et al., 2002b, 2016), and the sensorial, nutritional, and technological traits of their meat are certainly different from those of the commercial genotypes (Castellini et al., 2002b; Fanatico et al., 2005), which can be valued by consumers. Additionally, these animals are valuable source of biodiversity in crosses with commercial genotypes (Soleimani et al., 2011). However, data on their performance are scarce (Soglia et al., 2020; Cartoni Mancinelli et al., 2023); with few studies are available about their response under challenging environmental conditions (Deeb and Cahaner, 2001), whereas several reports have been published about the effects of heat stress in standard commercial genotypes (Brugaletta et al., 2022). This lack of information is especially relevant for Italian local breeds. Among these latter, Bionda Piemontese (**BP**) and Robusta Maculata (**RM**) are slow-growing chickens, originating from Piedmont (north-west of Italy) and Veneto (north-east of Italy), respectively, which could be of interest for meat and egg production (Ferrante et al., 2016).

Thus, the present study aimed to determine the response of different genotypes, 1 commercial crossbreed (Ross 308) and 2 Italian local breeds (BP and RM) under different environmental conditions (standard vs. high temperature), as to their productive performance, carcass traits, and meat rheological, nutritional, and sensorial properties.

## MATERIALS AND METHODS

### Ethics Statement

The study was approved by the Ethical Committee for Animal Experimentation (Organismo Preposto al Benessere degli Animali, **OPBA**) of the University of Padova, Italy (project 7/2021; Prot. n. 15481, approved on 01/02/2021). All animals were handled according to the principles stated in the EC Directive 2010/63/EU. Research staff involved in animal handling were animal specialists (PhD or MS in Animal Science) and veterinary practitioners.

### Facilities, Animals, Diets, and In Vivo Recordings

The trial was performed at the poultry house of the University of Padova (Legnaro, Padova, Italy), during the period from October to March. The poultry house had 2 identical rooms, both equipped with a cooling system, forced ventilation, radiant heating and controlled light systems. A data logger (P5185, PeakTech, Prüf- und Messtechnik GmbH Gerstenstieg, Ahrensburg, Germany) was placed in the center of each room, approximately at 30 cm above the ground, to record temperature and relative humidity during 24 h.

A total of 24 wire-net pens (1.25 m wide × 2.60 m long × 1.20 m height; 3.25 m^2^ total surface; 12 pens per room) were used. Each pen was equipped with 5 automatic nipple drinkers and a circular feeder (diameter: 37 cm) for manual distribution of feed. The pens had a concrete floor covered with litter made of a 50 to 50 mixture of wood shavings and chopped wheat straw (depth 7 cm, 2.5 kg/m^2^). Twenty-four hours of light were provided during the first 2 d after the chickens arrived at the poultry house. Then, the hours of lights were progressively reduced until a 18L:6D light program was reached, which was maintained from the 12th d onward.

A total of 238 two-days-old chicks of both sexes belonging to 3 genotypes were used, that is, 105 chicks of a commercial fast-growing genetic line, Ross 308 (Aviagen Group, Newbridge, UK) (52 females, 53 males); 100 chicks Bionda Piemontese (56 females, 44 males); and 33 chicks of Robusta Maculata (15 females, 18 males) were used. The different numbers of chicks among genotypes and the unbalanced sex ratio for local breeds were due to the low hatching performance of the local breeds, especially RM. All eggs of the 3 genotypes were incubated under the same conditions in the same commercial hatchery. Eggs of Ross chickens came from a standard commercial breeding farm; eggs of BP and RM chickens were produced at the experimental facilities of the University of Torino and the University of Perugia, respectively. For these 2 local breeds, the incubated eggs were 150 for BP and 196 for RM; the fertile eggs were 144 and 47 for BP and RM, respectively; and the hatched eggs were 100 and 33 for BP and RM. Notwithstanding the higher number of incubated eggs of RM, due to the low fertility and hatching performance, the final number of RM chicks available for the trial was lower than what scheduled (around 50 per genotype per sex). All chicks were vaccinated against coccidiosis, Marek's disease, infectious bronchitis, and Newcastle disease at the hatchery, and delivered by a commercial authorized truck to the experimental facilities of the University of Padova.

On their arrival, the chicks were individually identified by a leg mark and randomly allocated to the 24 pens of the 2 rooms according to a tri-factorial arrangement, that is, 3 genotypes (Ross 308, BP, and RM), 2 room temperatures (standard, S vs. high, H temperature), and 2 sexes (i.e., 12 experimental groups, 2 replicates per group) until slaughtering (42 d of age for Ross 308 and 99 d of age for RM and BP chickens). According to the total number of chicks available, the number of birds per pen was 13 to 14 for Ross, 12 to 14 for BP, and 4 to 5 for RM chickens.

As for the environmental temperature, the range used in the room under standard conditions (S) was set according recommendations for broiler chickens until the fifth week of age (Aviagen, 2018), that is, decreasing from 28°C to 30°C during the first week to 20°C to 22°C during fifth week onward. In the room with high temperature (H), artificial heating was set to maintain a higher temperature of 1°C to 2°C during the first week and then increasing the difference until 6°C to 7°C, to provoke a moderate heat stress, but always in the acceptable range of temperature. Then, the average difference in the mean 24-h temperature between the 2 rooms was on average +4.7°C, as detailed in Table 1).

**Table 1:** Summary of temperature and relative humidity conditions in different rooms, standard (S) vs. high (H) temperature from 1 to 14 wk of age.

Week	Room	Temperature, °C			Relative humidity, %		
		24-h average	Maximum	Minimum	24-h average	Maximum	Minimum
Wk 1	S	29.0 ± 0.7	29.7 ± 0.5	28.2 ± 0.8	40.3 ± 1.0	42.8 ± 2.2	38.0 ± 1.0
	H	29.9 ± 0.5	31.2 ± 1.0	28.8 ± 0.8	39.2 ± 0.9	41.6 ± 1.9	37.4 ± 1.0
DIFF	S vs. H	+0.9	+1.5	+0.6	−1.1	−1.2	−0.6
Wk 2	S	27.3 ± 0.4	28.0 ± 0.9	26.8 ± 0.3	37.0 ± 1.4	39.4 ± 1.9	34.2 ± 1.3
	H	29.2 ± 0.3	30.3 ± 0.4	28.3 ± 0.2	34.4 ± 1.0	36.8 ± 1.4	32.0 ± 1.0
DIFF	S vs. H	+1.9	+2.3	+1.6	−2.6	−2.6	−2.2
Wk 3	S	26.2 ± 0.5	26.8 ± 0.5	25.6 ± 0.5	41.5 ± 1.4	44.0 ± 1.8	38.3 ± 1.4
	H	29.0 ± 0.7	29.8 ± 0.6	27.8 ± 0.8	36.6 ± 1.2	38.9 ± 2.2	34.6 ± 0.8
DIFF	S vs. H	+2.7	+3.1	+2.2	−5.0	−5.1	−3.7
Wk 4	S	25.1 ± 0.3	26.1 ± 0.4	23.9 ± 0.4	34.0 ± 2.9	37.2 ± 4.4	29.8 ± 2.8
	H	27.6 ± 0.5	29.1 ± 0.7	25.6 ± 0.6	31.5 ± 2.3	33.7 ± 3.7	29.1 ± 1.9
DIFF	S vs. H	+2.4	+3.0	+1.7	−2.5	−3.5	−0.7
Wk 5	S	23.7 ± 0.8	24.2 ± 0.6	23.1 ± 1.3	36.1 ± 2.6	40.9 ± 9.1	33.6 ± 3.0
	H	27.7 ± 0.7	28.5 ± 0.8	26.5 ± 0.8	31.8 ± 2.5	33.3 ± 1.9	30.2 ± 2.7
DIFF	S vs. H	+3.9	+4.3	+3.5	−4.3	−7.6	−3.4
Wk 6	S	22.4 ± 0.8	23.1 ± 0.8	21.6 ± 0.9	44.5 ± 4.4	48.8 ± 5.1	40.1 ± 4.7
	H	26.8 ± 1.5	28.0 ± 1.6	25.4 ± 2.0	36.5 ± 2.4	39.1 ± 3.1	34.1 ± 2.3
DIFF	S vs. H	+4.3	+4.9	+3.8	−8.1	−9.7	−6.0
Wk 7	S	21.8 ± 0.4	22.5 ± 0.3	21.0 ± 0.5	37.0 ± 2.3	40.5 ± 1.0	32.8 ± 3.2
	H	25.8 ± 0.9	27.4 ± 0.9	23.8 ± 1.2	30.7 ± 1.7	33.3 ± 1.0	28.6 ± 1.8
DIFF	S vs. H	+4.0	+4.8	+2.8	−6.2	−7.2	−4.2
Wk 8	S	21.9 ± 0.2	22.4 ± 0.3	21.1 ± 0.2	44.1 ± 2.4	45.7 ± 2.4	41.8 ± 3.5
	H	27.0 ± 0.4	28.1 ± 0.3	25.5 ± 0.5	35.3 ± 1.0	37.3 ± 0.9	33.8 ± 1.5
DIFF	S vs. H	+5.1	+5.7	+4.4	−8.8	−8.4	−8.0
Wk 9	S	20.8 ± 0.3	21.5 ± 0.5	19.8 ± 0.2	35.2 ± 4.3	38.7 ± 4.4	31.8 ± 4.1
	H	24.4 ± 0.9	26.0 ± 1.1	22.3 ± 0.9	31.2 ± 2.8	34.0 ± 1.8	29.1 ± 3.2
DIFF	S vs. H	+3.6	+4.5	+2.5	−4.0	−4.7	−2.7
Wk 10	S	20.5 ± 0.6	21.0 ± 0.7	19.9 ± 0.8	40.6 ± 1.9	43.1 ± 2.1	37.3 ± 2.4
	H	25.4 ± 0.8	26.8 ± 0.9	23.1 ± 0.9	33.7 ± 1.2	35.2 ± 1.7	31.6 ± 1.5
DIFF	S vs. H	+4.9	+5.8	+3.2	−6.9	−7.9	−5.7
Wk 11	S	19.9 ± 0.2	20.5 ± 0.2	19.1 ± 0.4	28.9 ± 1.9	30.6 ± 2.4	26.8 ± 1.0
	H	25.7 ± 0.6	27.7 ± 0.6	23.1 ± 1.0	24.1 ± 1.0	25.5 ± 1.6	22.6 ± 0.9
DIFF	S vs. H	+5.8	+7.3	+3.9	−4.8	−5.1	−4.2
Wk 12	S	20.0 ± 0.2	20.5 ± 0.1	19.3 ± 0.3	31.1 ± 5.2	32.9 ± 6.1	28.4 ± 3.8
	H	25.7 ± 0.7	27.5 ± 0.6	23.3 ± 1.1	25.5 ± 3.0	27.1 ± 3.4	24.0 ± 2.5
DIFF	S vs. H	+5.7	+6.9	+4.1	−5.6	−5.8	−4.3
Wk 13	S	20.5 ± 0.4	21.0 ± 0.5	19.8 ± 0.5	35.6 ± 7.6	38.8 ± 7.8	32.2 ± 7.3
	H	26.3 ± 1.1	28.0 ± 1.0	24.0 ± 1.7	28.3 ± 3.8	30.0 ± 3.9	26.7 ± 3.9
DIFF	S vs. H	+5.8	+7.0	+4.2	−7.2	−8.7	−5.5
Wk 14	S	21.7 ± 0.6	23.3 ± 0.4	20.7 ± 0.4	42.4 ± 9.5	49.5 ± 13.7	35.4 ± 6.6
	H	26.7 ± 1.1	28.2 ± 1.2	24.5 ± 1.1	29.5 ± 2.5	31.9 ± 3.5	27.2 ± 2.3
DIFF	S vs. H	+4.9	+4.9	+3.8	−12.9	−17.6	−8.2

Two commercial diets in crumble form, produced by a commercial feed mill (Consorzio Agrario di Treviso e Belluno, Paese, TV, Italy), were fed to the chickens during the trial. The diet of the first period was fed until 23 d to Ross 308 chickens and until 42 d to RM and BP chickens. It contained (% as fed): crude protein 20.2%, ether extract 4.88%, crude fiber 1.13%, lysine 1.27%, methionine 0.58%, calcium 1.10%, phosphorus 0.72%. The diet of the second period was fed from 24 to 42 d to Ross-308 chickens and from 43 to 99 d to RM and BP chickens. It contained (% as fed): crude protein 19.3%, ether extract 4.60%, crude fiber 1.42%, lysine 1.06%, methionine 0.49%, calcium 0.90%, phosphorus 0.70%.

The animals were individually weighed the day of their arrival and, then, once a week until commercial slaughtering. Mortality and pen feed consumption were daily measured during the trial.

### Commercial Slaughtering and Carcass and Meat Quality Recordings

At 42 d of age for Ross 308 and 99 d of age for BP and RM, all chickens were slaughtered in a commercial slaughterhouse, after approximately 7 h of feed withdrawal and approximately 4 h of water withdrawal. Ready-to-cook carcasses were recovered after 2 h of refrigeration at 2°C, individually weighed to measure slaughter dressing yield and transported to the DAFNAE laboratories to be stored at 2°C for 24 h. Then, the carcasses were submitted to gross examination to evaluate the occurrence (presence or absence) and the degree (normal, moderate, severe) of white striping (**WS**) on *pectoralis major* (***p. major***) muscle (Kuttappan et al., 2012, 2013), and the occurrence (presence or absence) of wooden breast (**WB**) (Sihvo et al., 2014) and spaghetti meat (**SM**) (Baldi et al., 2018). Afterward, 126 carcasses (6 per pen for Ross 308 and BP; 3–4 per pen for RM) were dissected for the main cuts (breast, wings, thighs, and drumsticks) (Petracci and Baéza, 2011). The right *p. major* muscles were used for the analyses of meat rheological properties (i.e., pH, water retention, color, and shear force). A total of 72 right breast muscles (3 per pen; 6 per experimental group) were used to analyze meat proximate composition, fatty acid profile and lipid peroxidation. Moreover, the left *p. major* muscles were stored under a vacuum in plastic bags at −18°C until sensory analysis.

### Meat Rheological Analyses

The pH values of the *p. major* muscles were measured in triplicate on their ventral side with a pH meter (Basic 20, Crison Instruments Sa, Carpi, Italy) equipped with a specific electrode (cat. 5232, Crison Instruments Sa). The L*a*b* color indexes were measured in triplicate in the ventral side of the same muscles using a Minolta CM508 C spectrophotometer (Minolta Corp., Ramsey, NJ) (Petracci and Baéza, 2011). After measuring the pH and color indexes, 1 meat portion (8 cm × 4 cm × 3 cm) was separated from the cranial side of the *p. major*, parallel to the direction of the muscle fibers, and stored under a vacuum in plastic bags at −18°C until the meat analyses. After thawing, the meat portion was cooked (inside its plastic bag) in a water bath at 80°C until the internal temperature of 80°C was achieved and cooking losses were measured in this cut according to Petracci and Baéza (2011). After 40 min of cooling, a meat portion (4 cm × 2 cm × 1 cm) was separated from the cooked cut to assess the maximum shear force with a LS5 dynamometer (Lloyd Instruments Ltd., Bognor Regis, UK) using the Allo-Kramer (10 blades) probe (load cell: 500 kg; distance between the blades: 5 mm; thickness: 2 mm; cutting speed: 250 mm/min) (Mudalal et al., 2015).

### Meat Proximate Composition, Fatty Acid Profile, and Lipid Peroxidation Analyses

After sampling the meat portion for cooking losses, the residual right *p. major* muscles were individually minced using a Grindomix GM 200 (Retsch GmbH, Haan, Germany). An aliquot of fresh minced meat was analyzed for fatty acid (**FA**) composition; another aliquot was used to determine the thiobarbituric acid reactive substances (**TBARs**) as a lipid peroxidation marker; the remaining meat was freeze-dried, reground, and used to determine dry matter (934.01), ash (967.05), crude protein (2001.11), and ether extract (991.36) contents (AOAC, 2000).

For the determination of fatty acid profile, fat was extracted from fresh aliquots by accelerated solvent extraction (**ASER**, Dionex, Sunnyvale, CA, Application Note 334) using 2 extraction cycles with petroleum ether as a solvent at 125°C and 10.3 Mpa, a 6-min heating phase, and a 2-min extraction phase. Then, 10 mL of NaSO_4_ (0.47% in H_2_O) was added to extracted lipids. Samples were kept at 4°C for 30 min and supernatant (petroleum ether and lipids) was collected in another vial previously weighed. Dry evaporation in N_2_ stream (Genevac EZ-2, SP Industries, Warminster, PA) was applied; residual samples (extracted lipids in vials) were weighed before adding 2 mL of 2% H_2_SO_4_ in methanol (Christie, 1989). Vials were stored at 50°C in a heater overnight. Thereafter, hexane (1 mL hexane/20 mg lipids) and potassium bicarbonate 2% (5 mL) were added. Samples were centrifuged, stored at 4°C for 30 min, and supernatant sampled to be analyzed by an Agilent 7820A Gas Chromatograph (Agilent Technologies, Santa Clara, CA), with a split to 92.199 mL/min and rate set at 65:1. Supelco OMEGAWAX-TM 250 (Sigma-Aldrich, St. Louis, MO) (30 m × 0.25 mm internal diameter, 0.25 μm film thickness) was used with hydrogen as the carrier at 1.4 mL/min. The oven temperature was set at 50°C, held for 2 min, raised to 220°C at the rate of 4°C/min, and then held for 17 min. Both the injector and the detector temperatures were set at 250°C. The individual FA were identified by comparing the retention time of the standard FA methyl esters mixture (Supelco 37-component FAME Mix, 47,885-U). Individual FA methyl esters were expressed as the percentage of the total area of eluted FA methyl esters.

The TBARs were evaluated according to Botsoglou et al. (1994), using spectrophotometric measurements (Jasco Mod. 7800 UV/VIS, Hachioji, Tokyo, Japan) at 532 nm, and data were expressed as mg of malondialdehyde (**MDA**)/kg.

### Meat Sensory Evaluation

The *p. major* muscles were evaluated by quantitative descriptive analysis (**QDA**) by a trained sensory panel to evaluate the differences in 11 descriptors. Each descriptor was evaluated on a structured continuous line scale with anchor points at 0 (not intense) and 10 (very intense). Twelve people (6 men and 6 women aged between 23 and 60) were selected out of the trained sensory panelists of the department DAFNAE according to ISO standards (ISO 8586, 3972, 5496). The panelists underwent a 1-month training period. In the first training phase, they got familiar with the product, generated, and agreed on attributes testing different chicken breasts prepared with different cooking methods to provide panelists with a wide range of diversity for each attribute. Once the panel became sufficiently familiar with the specific vocabulary, and the evaluation protocol was established, the panelists conducted a second phase of training, focusing on quantitative evaluation to acquire familiarity with the use of the intensity scale (range 0–10). Assessors were asked not to smoke, eat, or drink anything for at least 1 h before the tastings. Over a period of 2 wk, panelists evaluated the chicken breasts in 4 sessions. All sessions took place at 10 am at the sensory tasting facility of DAFNAE. In each session, each panelists evaluated 2 sets of 3 chicken breasts (each belonging to a different experimental group), with 15-min break between the 2 sets. Thus, during the 4 sessions, each panelist evaluated a total of 24 breast samples (2 per experimental group). The assessors were asked to evaluate 1 chicken breast per time, starting from the odor attributes (brothy; chickeny/meaty, wet feathers) followed by the taste (sweet; salty) and texture (cohesiveness; hardness; juiciness; chewiness; toothpack) attributes, and finally, the general liking (pleasantness). After each sample, panelists cleaned their palate with a piece of apple, an unsalted cracker, and some water, and after a 2-min break, they continued with the next sample. Each sample tested by the panelists consisted of 1 piece of cooked chicken breast (3 cm × 2 cm × 1 cm).

To obtain the samples for tasting, the chicken breasts were defrosted overnight at 4°C. The breasts of Ross-308 chickens were cut in pieces of 7 cm × 8 cm with 1.5 cm thickness; those of RM and BP chickens were cooked as whole being smaller in size. The meat pieces were sealed in bags under vacuum and cooked in a water bath at a constant temperature of 85°C for 25 min. Thereafter, each meat piece was divided in 4 equal samples (3 cm × 2 cm × 1 cm) and immediately served to the panelists. Presentation order was systematically varied by means of a Williams Latin square design, in order to balance the effects of the serving order and carryover effect. Data were collected using Fizz v2.47b software program (Biosystemes, Couternon, France).

### Statistical Analyses

Individual data of live weight, daily growth, and carcass traits were analyzed by analysis of variance (**ANOVA**), using the PROC MIXED of SAS software (SAS Institute Inc., 2013). The model included genotype, room temperature, and sex, as main factors of variability with interactions, and the pen as a random effect; the initial live weight was included as a covariate. Pen data of feed intake and feed conversion ratio were analyzed by ANOVA with the same main factors using the PROC GLM (SAS Institute Inc., 2013). The occurrence of myopathies in Ross chickens (myopathies were not detected in BP and RM chickens) at commercial slaughtering was analyzed with the PROC CATMOD (SAS Institute Inc., 2013), according to room temperature, sex, and their interactions. Results of sensory meat analyses were processed using the PROC MIXED of SAS (SAS Institute Inc., 2013) with a model including genotype, room temperature, sex, and their interactions as main factors of variability, and the panelist as a random effect. Differences among the means with *P* ≤ 0.05 were accepted as statistically significant. The Bonferroni *t* test was used to compare means.

## RESULTS

### Growth Performance

During the trial 6 chickens died (1 male Ross 308; 2 males BP and 3 males RM), thus, the mortality rate averaged 2.5% without significant differences among groups.

For final weight and daily weight gain, there were significant effects of genotype, temperature, and sex (both, *P* < 0.001) (Table 2) as well as significant interactions between the different factors (*P* < 0.001). As for the interaction genotype × temperature (Table S1), at both temperatures, the Ross 308 birds had the highest final weight and daily weight gain, while RM had higher final live weight and daily weight gain compared to the BP birds (all, *P* < 0.05). The final live weight and daily weight gain were lower at high temperature for both the Ross 308 and BP birds (*P* < 0.05), while similar values were recorded for the RM birds at both temperatures.

**Table 2:** Growth performance (LS means) of Ross 308, Bionda Piemontese (BP), and Robusta Maculata (RM) chickens reared under standard or high room temperature in separated sexes.

	Genotype (G)			Room temperature (T)		Sex (S)		*P* value			RMSE
Variables	Ross 308	BP	RM	Standard	High	Female	Male	G	T	S	
Chickens, n	104	98	30	115	117	123	109				
Initial live weight^1^, g	40.0^a^	39.2^a^	36.7^b^	38.9	38.3	38.8	38.4	<0.001	0.236	0.467	3.3
Final live weight^2^, g	2,852^a^	1,771^c^	2,216^b^	2,517	2,043	1,999	2,561	<0.001	<0.001	<0.001	213
Daily weight gain^3^, g/d	70.2^a^	20.0^c^	25.5^b^	43.8	33.4	34.4	42.8	<0.001	<0.001	<0.001	4.3
Feed intake^4^, g/d	104.4^a^	58.1^b^	62.7^b^	80.1	70.0	67.8	82.3	<0.001	<0.001	<0.001	5.1
Feed conversion	1.59^c^	3.56^a^	3.08^b^	2.77	2.71	2.84	2.64	<0.001	0.452	<0.01	0.13

Abbreviation: RSME, root mean square error.

1 Live weight at 2 d of age for all genotypes.

2 Live weight at 42 d of age for Ross 308; 99 d of age for BP and RM.

3 Individual data.

4 Pen data.

a,b,c Values with different superscript letters within the same line and effect are significant different (*P* < 0.05).

As for the significant interaction genotype × sex (*P* < 0.001) (Table S2), the daily weight gain of RM females was similar to that to that of BP males. Then, as for the significant interaction temperature × sex (*P* < 0.001) (Table S3), the final live weight and daily weight gain of males kept at higher temperature was similar to females kept at standard temperature (Table S3).

Then, as for the main effect of genotype, the Ross chickens showed the highest feed intake and the best feed conversion compared to the local breeds (*P* < 0.001) (Table 2), whereas the RM chickens achieved a lower feed conversion (−13.5%; *P* < 0.001) compared to BP chickens (Table 2). As for the temperature effect, the increase of temperature lowered daily feed intake (−12.6%; *P* < 0.001), and final live weight (−18.8%; *P* < 0.001), without affecting feed conversion (*P* > 0.10). Finally, as for the effect of sex, males showed a higher feed intake (+17.6%; *P* < 0.001) and lower feed conversion (−7.0%; *P* < 0.01) compared to females (Table 2).

### Slaughter Results

Consistently with final live weight, for carcass weight and traits there were significant effects of genotype, temperature, and sex (both, *P* < 0.001) (Table 3) as well as significant interactions between the different factors (*P* < 0.001). As for the interaction genotype × temperature (Table S1), changes in cold carcass weight were consistent with those reported for final live weight. Moreover, when the temperature of the room increased, the breast yield and the *p. major* proportion decreased, whereas the hind legs proportion increased only in Ross chickens (Table S1). Despite statistically significant, the interactions recorded between genotype and sex for slaughter results and carcass traits were not relevant (Table S2). Finally, as for carcass weight, there was a significant interaction between temperature and sex (*P* < 0.001), as males kept at a higher temperature had a carcass weight similar to females kept at a standard temperature (Table S3).

**Table 3:** Slaughter results and carcass traits (LS means) of Ross 308, Bionda Piemontese (BP), and Robusta Maculata (RM) chickens reared under standard or high room temperature in separated sexes.

	Genotype (G)			Room temperature (T)		Sex (S)		*P* value			RMSE
Variables	Ross 308^1^	BP^2^	RM^2^	Standard	High	Female	Male	G	T	S	
Chickens, n	48	48	30	63	63	63	63				
Cold carcass weight, g CC	2,132^a^	1,222^c^	1,561^b^	1,800	1,477	1,439	1,837	<0.001	<0.001	<0.001	156
Slaughter yield, %	74.9^a^	67.9^c^	69.8^b^	70.2	71.5	70.9	70.9	<0.001	<0.01	0.907	1.95
Dissected carcasses, n	48	48	30	63	63	63	63				
Breast yield^3^, % CC	40.4^a^	23.5^c^	28.0^b^	31.0	30.2	31.4	29.8	<0.001	<0.05	<0.001	1.74
*Pectoralis major*, % CC	26.3^a^	10.5^c^	13.4^b^	17.1	16.4	17.0	16.5	<0.001	<0.05	0.146	1.78
Wings, % CC	9.9^b^	12.6^a^	12.0^a^	11.3	11.5	11.8	11.1	<0.001	0.293	0.005	1.35
Thighs, % CC	15.3^b^	17.8^a^	18.0^a^	17.1	17.0	16.6	17.4	<0.001	0.732	0.001	1.25
Drumsticks, % CC	12.8^b^	15.4^a^	15.6^a^	14.5	14.8	13.9	15.3	<0.001	<0.05	<0.001	0.80
Hind legs, % CC	28.1^b^	33.2^a^	33.6^a^	31.5	31.8	30.6	32.7	<0.001	0.365	<0.001	1.39

Abbreviation: RSME, root mean square error.

1 Slaughtered at 42 d of age.

2 Slaughtered at 99 d of age.

3 With bone and skin.

a,b,c Values with different superscript letters within the same line and effect are significant different (*P* < 0.05).

As for the main effect of the room temperature, chickens kept at higher temperatures showed higher slaughter yield (+1.9%; *P* < 0.01) compared to chickens kept at standard temperatures. The proportions of breasts and *p. major* decreased with increasing the room temperature (−2.6% and −4.1%, respectively; *P* < 0.05), whereas that of drumsticks increased (+2.1%; *P* < 0.05) (Table 3).

As for the main effect of sex, male chickens showed higher proportions of thighs (+4.8%), drumsticks (+10.1%), and hind legs (+6.7%) compared to females (*P* < 0.001), and a lower proportion of breast yield (−5.1%; *P* < 0.001) and wings (−5.9%; *P* < 0.001) (Table 3).

### Meat Quality

At the *p. major* muscle, Ross chickens showed higher pH (+2.2% on average), lightness (+11.1%), yellowness (+7.2%), compared to the local breeds, whereas BP showed the highest redness (*P* < 0.001) (Table 4). Moreover, Ross meat had significantly higher water (+4.8%) contents, and a 3-fold increase in the ether extract content compared to BP and RM chickens (*P* < 0.001) (Table 4). Males presented higher ultimate pH values at breast than females (+2.2%; *P* < 0.001), which corresponded to lower lightness and yellowness indexes (−2.2% and −8.7%, respectively; 0.01 < *P* < 0.001). Meat of males had also significantly higher water and lower crude protein contents compared to the latter ones (*P* < 0.05) (Table 4).

**Table 4:** Rheological traits, lipid oxidation status (TBARs), and chemical composition of the *pectoralis major* muscle of Ross 308, Bionda Piemontese (BP), and Robusta Maculata (RM) chickens reared under standard or high room temperature in separated sexes.

	Genotype (G)			Room temperature (T)		Sex (S)		*P* value			RMSE
Variables	Ross 308^1^	BP^2^	RM^2^	Standard	High	Female	Male	G	T	S	
*Pectoralis major*, n	48	48	30	63	63	63	63				
pH	5.98^a^	5.83^b^	5.87^b^	5.89	5.90	5.83	5.96	<0.01	0.754	<0.001	0.19
L*	53.0^a^	46.7^b^	47.5^b^	48.7	49.4	49.8	48.7	<0.001	0.163	<0.01	2.44
a*	0.76^b^	1.93^a^	0.86^b^	1.19	1.17	1.10	1.26	<0.001	0.903	0.295	0.82
b*	13.9^a^	13.2^b^	12.6^b^	13.3	13.2	13.8	12.6	<0.001	0.602	<0.001	1.37
Cooking losses, %	41.3^a^	33.1^b^	32.9^b^	36.0	35.5	35.5	36.0	<0.001	0.557	0.502	4.19
Shear force, kg/g	4.65	6.09	4.88	4.89	5.52	4.68	5.74	0.328	0.487	0.243	4.95
Breast muscles, n	24	24	24	36	36	36	36				
TBARs, mg MDA/kg	0.10	0.16	0.16	0.13	0.15	0.15	0.13	0.146	0.527	0.552	0.09
Dry matter, %	22.4^b^	26.2^a^	26.1^a^	24.9	24.9	25.2	24.6	<0.001	0.800	<0.05	1.03
Water, %	77.6^a^	73.8^b^	73.9^b^	75.1	75.1	74.8	75.4	<0.001	0.800	<0.05	1.04
Crude protein, %	19.2^b^	24.3^a^	24.3^a^	22.7	22.5	22.9	22.3	<0.001	0.606	<0.05	0.97
Ether extract, %	2.24^a^	0.75^b^	0.65^b^	1.14	1.28	1.16	1.27	<0.001	0.188	0.307	0.43
Ash, %	1.07^b^	1.14^a^	1.15^a^	1.12	1.12	1.13	1.11	<0.001	0.934	0.017	0.03

Abbreviation: RSME, root mean square error.

1 Slaughtered at 42 d of age.

2 Slaughtered at 99 d of age.

a,b Values with different superscript letters within the same line and effect are significant different (*P* < 0.05).

As for cooking losses and crude protein contents, significant effects of genotype, sex, and their interactions were recorded (Table S2): meat cooking losses and crude protein contents differed between males and females of Ross chickens, whereas they were similar between sexes for local breeds (*P* < 0.05).

No effect of the room temperature was recorded on meat quality traits (Table 4).

### Occurrence of Breast Myopathies

Breast myopathies occurred only in Ross chickens where males showed a higher rate of WB (+19.4 percentage units; *P* < 0.001) and a lower rate of SM (−12.6 percentage units; *P* < 0.01) compared to females (Table 5). When the room temperature increased, the rate of breasts affected by WS (whatever degree) and SM decreased (−11.6 and −10.8 percentage units, respectively; 0.05 < *P* < 0.01) (Table 5).

**Table 5:** Myopathy rates (means) at gross examination in Ross 308 chickens reared under standard or high room temperature in separated sexes.

	Room temperature (T)		Sex (S)		*P* value	
Variables	Standard	High	Female	Male	T	S
Carcasses, n	52	52	52	52		
Moderate white striping, %	24.3	16.5	21.4	19.4	0.092	0.750
Severe white striping, %	14.5	10.7	9.7	15.5	0.334	0.156
Total white striping^1^, %	38.8	27.2	31.1	34.9	0.01	0.332
Wooden breast^1^, %	13.6	21.4	7.8	27.2	0.114	<0.001
Spaghetti meat^1^, %	21.4	10.6	22.3	9.7	<0.05	0.01

1 Not exclusive myopathy, that is, white striping, wooden breast, and/or spaghetti meat can be associated in the same breast.

### Fatty Acid Profile and Lipid Peroxidation

Breast muscles showed a similar profile among genotypes as for the whole saturated (**SFA**), monounsaturated (**MUFA**), and polyunsaturated fatty acids (**PUFA**) proportions (Table 6). Within SFA, Ross chickens exhibited a lower rate of stearic acid (C18:0) and other minor SFA than local breeds (*P* < 0.001). Within MUFA, Ross chickens showed a lower rate of palmitoleic (C16:1 n7) (*P* < 0.01) and a higher rate of oleic acid (C18:1 n9) (*P* < 0.001) compared to local breeds (Table 6). Moreover, males exhibited higher rate of total PUFA and n-6 PUFA (*P* < 0.01) with higher rates of linoleic (C18:2 n6) and α-linolenic (C18:3 n3) acids (0.05 < *P* < 0.01) compared to females (Table 6). Finally, as for the effect of the room temperature, chickens reared under high temperature exhibited a higher rate of α-linolenic (C18:3 n3) acid (*P* < 0.01) (Table 6).

**Table 6:** Fatty acids composition (% total FA) of breast fillets (*pectoralis major*) of Ross 308, Bionda Piemontese (BP), and Robusta Maculata (RM) chickens reared under standard or high room temperature in separated sexes.

	Genotype (G)			Room temperature (T)		Sex (S)		*P* value			RMSE
Variables	Ross 308	BP	RM	Standard	High	Female	Male	G	T	S	
Breast muscles, n	24	24	24	36	36	36	36				
C16:0	32.1	30.8	30.7	31.1	31.2	31.7	30.7	0.273	0.872	0.164	2.3
C18:0	4.00^b^	5.60^a^	5.49^a^	5.03	5.04	4.95	5.11	<0.001	0.978	0.567	0.91
Other SFA	0.35^a^	0.59^a^	0.72^a^	0.52	0.58	0.53	0.57	<0.001	0.304	0.548	0.19
C16:1 n7	1.44^b^	3.39^a^	3.22^a^	2.92	2.45	2.88	2.49	0.01	0.334	0.428	1.56
C18:1 n9	31.3^a^	27.5^b^	26.3^b^	28.5	28.2	29.0	27.8	<0.001	0.659	0.116	2.37
C18:1 n7	1.20	1.53	1.52	1.61	1.22	1.49	1.33	0.339	<0.05	0.397	0.59
Other MUFA	1.11	1.00	1.09	1.14	0.99	1.07	1.06	0.601	0.120	0.865	0.31
C18:2 n6	25.8	27.0	27.9	26.2	27.6	25.7	28.1	0.105	0.082	<0.01	2.4
C18:3 n3	1.67^a^	1.39^b^	1.54^a^	1.46	1.60	1.48	1.59	<0.001	<0.01	<0.05	0.14
C20:5 n3	0.037	0.044	0.102	0.073	0.048	0.072	0.049	0.293	0.516	0.558	0.123
C22:6 n3	0.002	0.045	0.034	0.029	0.025	0.033	0.022	<0.01	0.636	0.208	0.028
Other PUFA	0.93	1.20	1.31	1.30	0.99	1.10	1.19	0.179	0.055	0.553	0.49
Σ SFA	36.4	37.0	36.9	36.7	36.9	37.2	36.4	0.880	0.825	0.336	2.67
Σ MUFA	35.1	33.4	32.2	34.2	32.9	34.4	32.7	0.065	0.161	0.070	3.01
Σ PUFA	28.5	29.6	30.9	29.1	30.3	28.4	31.0	0.086	0.180	<0.01	2.72
Σ UFA	63.6	63.0	63.1	63.3	63.1	62.8	63.6	0.879	0.824	0.335	2.67
Σ n3	1.71^a^	1.48^b^	1.67^a^	1.56	1.68	1.58	1.66	0.01	0.083	0.241	0.21
Σ n6	26.6	27.8	28.8	27.2	28.4	26.5	29.0	0.121	0.141	<0.01	2.57

Abbreviations: MUFA, monounsaturated FA; PUFA, polyunsaturated FA; RSME, root mean square error; SFA, saturated FA; UFA, unsaturated FA.

a,b Values with different superscript letters within the same line and effect are significant different (*P* < 0.05).

### Sensory Analysis of Broiler Breast

At the sensory analysis, there were significant effects of genotype and temperature (both, *P* < 0.001) (Table 7) as well as significant interactions between the 2 factors (*P* < 0.001) for texture: cohesiveness, hardness, chewiness, and toothpack scores increased, and juiciness score decreased in chickens kept under high temperature only in Ross chickens without differences between local breeds kept in the 2 rooms (Table S1).

Then, as for flavor, the meat of Ross chickens received a lower score for “brothy and chickeny/meaty” and a higher one for “wet feathers” compared to meat of local breeds (0.05 < *P* < 0.001).

Finally, as for differences between sexes, breasts from males were scored higher for hardness (+2.8%; *P* < 0.001), juiciness (+4.2%; *P* < 0.001), and chewiness (+0.9%; *P* < 0.01), besides for a higher salty flavor (+2.0%; *P* < 0.01) (Table 7).

**Table 7:** Sensory mean scores (0–10 scale) of descriptive attributes of cooked breast fillets ( *pectoralis major*) of Ross 308, Bionda Piemontese (BP), and Robusta Maculata (RM) chickens reared under standard or high room temperature in separated sexes.

	Genotype (G)			Room temperature (T)		Sex (S)		*P* value			RMSE
Variables	Ross 308	BP	RM	Standard	High	Female	Male	G	T	S	
Chickens, n	24	24	24	36	36	36	36				
Texture											
Cohesiveness	3.32	3.32	3.79	3.35	3.60	3.49	3.47	0.081	0.910	0.212	1.67
Hardness	4.52	4.29	4.19	4.02	4.65	4.27	4.39	0.271	0.487	<0.001	1.47
Juiciness	5.46^a^	4.78^b^	4.92^ab^	5.33	4.78	4.95	5.16	<0.05	0.268	<0.01	1.63
Chewiness	4.23	4.48	4.26	4.15	4.62	4.37	4.41	0.563	0.819	<0.01	1.73
Toothpack	3.75	3.64	3.84	3.68	3.80	3.93	3.55	0.690	<0.05	0.522	1.59
Flavor/taste											
Brothy	3.88^b^	4.54^a^	4.30^a^	4.26	4.22	4.31	4.17	<0.05	0.846	0.452	1.52
Chickeny/meaty	5.92^b^	6.48^a^	6.45^a^	6.29	6.27	6.26	6.30	<0.001	0.852	0.734	1.07
Wet feathers	5.74^a^	4.46^b^	4.74^a^	4.96	5.00	4.86	5.09	<0.001	0.858	0.242	1.67
Sweet	6.20	6.38	6.31	6.38	6.21	6.30	6.29	0.496	0.947	0.172	1.06
Salty	5.03	5.08	5.05	5.25	4.86	5.00	5.10	0.949	0.407	<0.01	1.03
Pleasantness	6.15	5.84	5.90	6.12	5.80	5.79	6.13	0.386	0.08	0.104	1.67

Abbreviation: RSME, root mean square error.

a,b Values with different superscript letters within the same line and effect are significant different (*P* < 0.05).

## DISCUSSION

Commercial crossbred chickens complete their productive cycle in less than 45 d compared local pure breeds who need from 70 to 100 d to meet an overall final live weight between 2.0 and 2.5 kg (Gordon and Charles, 2002; Fanatico et al., 2005; Soglia et al., 2020). In the present trial, 99 d were not enough for BP chickens to reach 2.0 kg of final live weight, whereas it was for RM chickens. Indeed, to achieve the maturity, local breeds, like Kabir or RM, are supposed to need around 75 and 82 d of age, respectively, whereas Ross chickens require 56 d (Castellini et al., 2002c). The use of genetic resources from local breeds could allow the industry to accommodate consumer animal welfare and environmental concerns (Soglia et al., 2020), despite the lower performance, slaughter and carcass yields of these animals compared to the fast-growing genotypes (Castellini et al., 2002a,c; Dal Bosco et al., 2021). Indeed, slaughter traits (carcass weight and slaughter yield) are more favorable in fast-growing chickens, in which the proportions of breast and other commercial cuts are much higher with respect to the viscera (Havenstein et al., 1994; Rosa et al., 2007). Certainly, an older age at slaughter of local breeds affects the chemical composition of the meat by decreasing the protein content and increasing the fat content at the expenses of water (Wattanachant et al., 2004; Fanatico et al., 2007; Sirri et al., 2011; Dal Bosco et al., 2021). Nonetheless, in agreement with the present study, other authors observed that the genetic selection for growth rate favors greater fat deposition in fast-growing broilers (Leclercq, 1988; Castellini et al., 2002c).

In addition, slow-growing genotypes are generally slaughtered at older ages, which promote the accumulation of pigments in the meat (Berri et al., 2001; Baéza et al., 2002; Devatkal et al., 2019) resulting into higher redness while lower lightness and pH compared to fast-growing genotypes (Berri et al., 2001; Quentin et al., 2003). On the other hand, the higher antioxidant capacity of local breeds is closely related with their higher locomotory activity (Bizeray et al., 2000; Castellini et al., 2002c; Dal Bosco et al., 2021), likewise correlated with the lightness, yellowness and TBARs values (Dal Bosco et al., 2021). It is well known that the oxidation of meat decreases its shelf life and negatively affect its flavor (Lorenzo and Gómez, 2012; Domínguez et al., 2019; Dal Bosco et al., 2021). In the present study there were no differences in the TBARs value among genotypes, however, panelists reported the higher “wet feathers” off-flavor for breast from Ross chickens. The lipid content and fatty acid profile of lipids in the meat can affect the oxidative susceptibility of the meat (Maraschiello et al., 1999; Dalle Zotte et al., 2020). Ross chickens presented higher ether extract content compared to local breeds and, thus, our results suggest that meat from local breeds would be less susceptible to oxidation than meat from the fast-growing genotype. Regarding texture attributes, due to the lower maturity and hypertrophy, meat from fast-growing chickens usually presents poor cohesion that reduces the water holding capacity, capillarity and blood supply (Dal Bosco et al., 2021), which could affect the juiciness perception (Fanatico et al., 2005, 2007). In agreement with the present study, other authors reported lower pH and water content value for breasts from slow-growing chickens (Wattanachant et al., 2004; Berri et al., 2005; Fanatico et al., 2007). Furthermore, Castellini et al. (2002c) confirmed higher juiciness for Ross breasts compared to RM chickens, due to the higher level of fat and water content in the meat.

As for the occurrence of myopathies in slow-growing genotypes and local breeds, to our knowledge, no reports are available in literature. Indeed, the use of slow-growing strains has been proposed as a strategy to alleviate these disorders (Petracci et al., 2019). Moreover, previous studies have reported that increased BW and growth rates are associated to increased incidence and severity of muscle abnormalities in broiler chickens (Kuttappan et al., 2012; Lorenzi et al., 2014; Alnahhas et al., 2016; Santos et al., 2021), thus slow-growing strains are expected to be less affected by myopathies than fast-growing chickens. The last statement was confirmed by Dixon (2020) and Santos et al. (2021), who found greater incidence of WB and WS in fast-growing compared to slow-growing birds.

Sexual dimorphism is generally an intrinsic feature for all chicken breeds, however, some local breeds, such as Bianca di Saluzzo or BP, show a poor or inexistent sexual dimorphism (Soglia et al., 2020). On the other hand, consistently with our results, differences between sexes for RM and Ross chickens are widely recognized in terms of final live weight, feed intake, and daily gain (Castellini et al., 2002a; Soglia et al., 2020; Bongiorno et al., 2022), even though slow-growing genotypes require longer time to display differences (Soglia et al., 2020). Finally, males exhibit higher carcass yield, legs proportion while lower breast yield than females (Castellini et al., 2002a; Bongiorno et al., 2022; Bordignon et al., 2022). Previous meta-analyses of ours (Bordignon et al., 2022), using data from different trials with commercial fast-growing genotypes, found that males had higher meat pH compared to females, which was associated to higher water retention and a higher lightness index, as stated by other authors (Campo et al., 2020). Differences in pH between sexes of fast-growing chickens are related to breast weights, that is, a correlation of breast muscle weight with muscle glycogen (negative correlation) and final pH (positive correlation) (Le Bihan-Duval et al., 2008). As for slow-growing chickens, Fanatico et al. (2005) did not observe differences for the lightness nor the redness between sexes, whereas other authors found that males displayed lower yellowness compared to females (Fanatico et al., 2005; Bongiorno et al., 2022). Finally, as for differences in myopathies occurrence between sexes, the results of the present study confirm that in fast-growing genotypes the occurrence of WB is higher in males compared to females, whereas the occurrence of SM shows an opposite trend (Bordignon et al., 2022).

Selection for growth and production has declined the meat chicken's ability to cope with environmental temperatures out of the thermoneutral range, hence becoming susceptible to both, acute and chronic heat stress (Soleimani et al., 2011; Patael et al., 2019; Halevy, 2020; Zampiga et al., 2021). A chronic heat exposure significantly worsened feed intake, growth rate (without affecting feed efficiency), and meat quality in fast and slow-growing genotypes, in the present trial as well as in previous studies (Cahaner and Leenstra, 1992; Yunis and Cahaner, 1999; Deeb et al., 2002; Lu et al., 2007). In addition, it has been reported that a chronic high temperature exposure was able to reduce breast yield while increasing fat deposition, due to the reduction on the basal metabolism and physical activity, thus deteriorating the quality of the meat (Geraert et al., 1996; Aksit et al., 2006; Rosa et al., 2007; Zampiga et al., 2021). Regarding the chemical composition of the meat, Rosa et al. (2007) observed that high room temperatures decrease meat protein content and increase fat content, whereas in the present study no significant difference was detected according to the environmental temperature. As for meat quality, some authors (McKee and Sams, 1997; Lu et al., 2007) reported that chickens reared under chronic heat displayed breasts with higher yellowness and normal pH despite the stress, whereas no variation was observed in the present study. Regarding the fatty acid composition, El-Tarabany et al. (2021) demonstrated that prolonged heat stress increased concentration of saturated FA, while reduced concentrations of MUFA and PUFA of breasts from Ross chickens. In the present study the increase of the room temperature showed only minor effects on the meat fatty acid profile. Differences on the duration and extent of the heat stress could explain different results among studies.

As for the role of environmental temperature on different genotypes, Lu et al. (2007) studied the effect of chronic heat stress in 2 genetic lines, a commercial fast-growing and a local slow-growing. Under higher temperature, they reported larger reduction in BW (data expressed in g) and weight gain (g/d) in commercial broilers compared to the local ones, and the same interaction was confirmed in the present study. However, if we express the variations in relative terms (%) than in absolute weight (g), the differences among genotypes were strongly reduced or even disappeared: final live weights of Ross, BP, and RM chickens submitted to high temperature were lower by 816 g (−26%), 301 g (−15.6%) and 299 g (−12.5%) compared to their counterparts kept at standard temperature conditions, which corresponded to a decrease of daily weight gain by 19.4 g/d (−25.3%) for Ross, 5.3 g/d (−24,0%) for BP, and 5.2 g/d (−18.9%) for RM.

On the other hand, as in the present trial, Lu et al. (2007) observed a decreased in the breast muscle proportion in commercial chickens reared under hot conditions, while breast and leg proportions remained unchanged for local breeds. Rosa et al. (2007) reared fast-growing and slow-growing chickens under heat exposure and reported better carcass yield, while lower thighs, drumsticks and wings yields in fast-growing chickens. The same authors reported that breast yield in slow-growing broilers under heat exposure was not affected. Nevertheless, in commercial broilers there was a reduction in the breast yield due to the decrease on the feed intake when birds were exposed to high temperature. In the present study we observed the same interaction: the breast yield decreased in fast-growing broilers reared under higher temperature, whereas it remained unchanged in local breeds.

## CONCLUSIONS

Local chicken breeds were confirmed to be less productive than commercial crossbreeds either in terms of growth performance or slaughter yields. On the other hand, the better meat quality traits of BP and RM breeds compared to Ross 308 can be valorized on the market and highly appreciated by the consumer both from a nutritional (high-protein and low-fat contents) and a sensorial (increase in positive flavors and decrease in off-flavors) point of view. Among local breeds, RM achieved better growth performance and slaughter yields compared to BP, thus being more sustainable.

High environmental temperatures greatly jeopardized growth performances and meat quality in Ross 308 chickens, whereas the effects on BP and RM were less pronounced especially in terms of carcass and meat quality. Therefore, the results of this study confirm that local chicken breeds represent a highly important genetic heritage that must be conserved to maintain biodiversity, and which could be exploited in genetic selection programs to improve resilience to environmental stresses.

## Appendix Supplementary materials

Image, application 1

## Footnotes

Supplementary material associated with this article can be found, in the online version, at doi:10.1016/j.psj.2023.103158.
