# Supplementary material for: Resiliency of fast-growing and slow-growing genotypes of broiler chickens submitted to different environmental temperatures: growth performance and meat quality
Source: Poult Sci. 2023 Oct 6;102(12):103158. doi: 10.1016/j.psj.2023.103158 (PMC10632536; doi:10.1016/j.psj.2023.103158)
Supplement: Supplementary file 1 [file mmc1.docx]

**Resiliency of fast-growing and slow-growing genotypes of broiler chickens submitted to different environmental temperatures: growth performance and meat quality**

Huerta A.^*^, Pascual A.^*^, Bordignon F.^*^, Trocino A.^*#1^, Xiccato G.^*^, Cartoni Mancinelli A.^§^, Mugnai C., Pirrone F.^*^, Birolo M.^*^

^*^Department of Agronomy, Food, Natural Resources, Animals and Environment (DAFNAE), University of Padova, Viale dell’Università 16, 35020 Legnaro, Padova, Italy

^#^Department of Comparative Biomedicine and Food Science (BCA), University of Padova, Viale dell’Università 16, 35020 Legnaro, Padova, Italy

^§^Department of Agricultural, Environmental, and Food Science, University of Perugia, Borgo XX Giugno 74, 06121 Perugia, Italy

Department of Veterinary Sciences, University of Turin, Largo Braccini 2, 10095 Grugliasco (Torino), Italy

^1^Corresponding author: angela.trocino@unipd.it

Angela Trocino, Department of Agronomy, Food, Natural Resources, Animals and Environment (DAFNAE), University of Padova, Viale dell’Università 16, 35020 Legnaro, Padova, Italy. Tel. +39 049 8272583; e-mail: [angela.trocino@unipd.it](mailto:angela.trocino@unipd.it)

Section: Metabolism and Nutrition

**SUPPLEMENTARY MATERIALS**

**Table S1.** Significant interactions “Genotype × Room temperature” in Ross 308, Bionda Piemontese (BP), and Robusta Maculata (RM) chickens.

| Room temperature (T) | Normal temperature | | |  | High temperature | | | |  | *P*-value |  | | RMSE |  |
| --- | --- | --- | --- | --- | --- | --- | --- | --- | --- | --- | --- | --- | --- | --- |
| Genotype (G) | Ross 308 | BP | RM |  | | Ross 308 | BP | RM |  | G × T |  | |  |  |
| Growth performance |  |  |  |  | |  |  |  |  |  |  |  | | |
| Final live weight, g | 3,256^a^ | 1,924^c^ | 2,390^b^ |  | | 2,440^b^ | 1,623^d^ | 2,091^c^ |  | <0.001 |  | 4.3 | | |
| Daily weight gain, g/d | 76.6^a^ | 22.1^d^ | 27.5^c^ |  | | 57.2^b^ | 16.8^e^ | 22.3^cd^ |  | <0.001 |  |  | | |
| Slaughter results and carcass traits | |  |  |  | |  |  |  |  |  |  |  | | |
| Cold carcass weight, g CC | 2,414^a^ | 1,329^d^ | 1,657^c^ |  | | 1,851^b^ | 1,115^e^ | 1,465^d^ |  | <0.001 |  | 156 | | |
| Breast yield, % CC | 41.6^a^ | 23.6^d^ | 27.9^c^ |  | | 39.3^b^ | 23.3^d^ | 28.0^c^ |  | <0.01 |  | 1.74 | | |
| Pectoralis major, % CC | 27.5^a^ | 10.5^d^ | 13.3^c^ |  | | 25.0^b^ | 10.6^d^ | 13.5^c^ |  | <0.01 |  | 1.78 | | |
| Thighs, % CC | 15.0^b^ | 18.0^a^ | 18.2^a^ |  | | 15.7^b^ | 17.6^a^ | 17.7^a^ |  | <0.05 |  | 1.25 | | |
| Hind legs, % CC | 27.5^c^ | 33.3^a^ | 33.8^a^ |  | | 28.7^b^ | 33.2^a^ | 33.4^a^ |  | <0.05 |  | 1.39 | | |
| Sensory analysis of *P. major* |  |  |  |  | |  |  |  |  |  |  |  | | |
| Cohesiveness | 2.79^b^ | 3.29^ab^ | 3.98^a^ |  | | 3.85^a^ | 3.35^ab^ | 3.60^ab^ |  | <0.05 |  | 1.67 | | |
| Hardness | 3.72^b^ | 4.17^b^ | 4.17^b^ |  | | 5.33^a^ | 4.41^b^ | 4.20^b^ |  | <0.001 |  | 1.47 | | |
| Juiciness | 6.07^a^ | 4.87^b^ | 5.04^b^ |  | | 5.33^b^ | 4.69^b^ | 4.85^b^ |  | <0.05 |  | 1.63 | | |
| Chewiness | 3.64^b^ | 4.43^ab^ | 4.39^ab^ |  | | 5.21^a^ | 4.54^ab^ | 4.12^b^ |  | <0.001 |  | 1.73 | | |
| Toothpack | 3.22^b^ | 3.75^ab^ | 4.08^ab^ |  | | 5.21^a^ | 3.54^ab^ | 4.27^ab^ |  | <0.01 |  | 1.59 | | |

RSME, root mean square error. ^a,b, c,d, e^ Values with different superscript letters within the same line and effect are significant different (P < 0.05).

**Table S2.** Significant interactions “Genotype × Sex” in Ross 308, Bionda Piemontese (BP) and Robusta Maculata (RM) chickens.

| Sex (S) | Females | | |  | Males | | |  | *P*-value |  | RSME |
| --- | --- | --- | --- | --- | --- | --- | --- | --- | --- | --- | --- |
| Genotype (G) | Ross 308 | BP | RM |  | Ross 308 | BP | RM |  | G × S |  |  |
| Growth performance |  |  |  |  |  |  |  |  |  |  |  |
| Daily weight gain, g/d | 61.0^b^ | 16.9^e^ | 21.3^d^ |  | 72.8^a^ | 22.0^d^ | 28.4^c^ |  | <0.001 |  | 4.3 |
| Slaughter results and carcass traits | |  |  |  |  |  |  |  |  |  |  |
| Breast yield, % CC | 40.9^a^ | 24.9^d^ | 28.4^c^ |  | 39.9^b^ | 22.0^e^ | 27.5^c^ |  | <0.01 |  | 1.74 |
| Wings, % CC | 9.6^c^ | 12.5^ab^ | 13.1^a^ |  | 9.6^c^ | 12.7^ab^ | 11.8^b^ |  | <0.01 |  | 1.35 |
| Drumsticks, % CC | 12.7^d^ | 14.2^c^ | 15.0^b^ |  | 12.9^d^ | 16.7^a^ | 16.2^a^ |  | <0.001 |  | 0.80 |
| Hind legs, % CC | 27.7^d^ | 31.3^c^ | 32.7^b^ |  | 28.5^d^ | 35.2^a^ | 34.5^a^ |  | <0.001 |  | 1.39 |
| Rheological traits and chemical composition of *P. major* | | |  |  |  |  |  |  |  |  |  |
| a* | 0.91^b^ | 1.60^ab^ | 0.91^b^ |  | 0.61^c^ | 2.27^a^ | 0.91^b^ |  | <0.05 |  | 0.82 |
| b* | 14.7^a^ | 14.1^a^ | 12.6^b^ |  | 13.1^ab^ | 12.3^b^ | 12.6^b^ |  | <0.05 |  | 1.37 |
| Cooking losses, % | 39.6^b^ | 33.8^c^ | 33.0^c^ |  | 44.2^a^ | 32.4^c^ | 32.7^c^ |  | <0.05 |  | 4.19 |
| Crude protein, % | 20.0^b^ | 24.4^a^ | 24.2^a^ |  | 18.4^c^ | 24.2^a^ | 24.3^a^ |  | <0.05 |  | 0.97 |

RSME, root mean square error. ^a, b, c, d, e^ Values with different superscript letters within the same line and effect are significant different (P < 0.05).

**Table S3.** Significant interactions “Room temperature × Sex” in Ross 308, Bionda Piemontese (BP) and Robusta Maculata (RM) chickens.

| Room temperature (T) | Normal temperature | |  | High temperature | |  | *P*-value |  | RMSE |
| --- | --- | --- | --- | --- | --- | --- | --- | --- | --- |
| Sex (S) | Females | Males |  | Females | Males |  | T × S |  |  |
| Growth performance |  |  |  |  |  |  |  |  |  |
| Final live weight, g | 2,184^b^ | 2,862^a^ |  | 1,825^c^ | 2,278^b^ |  | <0.001 |  | 213 |
| Daily weight gain, g/d | 37.0^b^ | 47.2^a^ |  | 29.2^c^ | 35.0^b^ |  | <0.001 |  | 4.3 |
| Slaughter results and carcass traits |  |  |  |  |  |  |  |  |  |
| Cold carcass weight, g CC | 1,569^b^ | 2,031^a^ |  | 1,310^c^ | 1,644^b^ |  | <0.01 |  | 156 |
| Wings, % CC | 11.7^a^ | 10.9^b^ |  | 11.9^a^ | 11.8^a^ |  | <0.05 |  | 1.35 |

RSME, root mean square error. ^a, b, c^ Values with different superscript letters within the same line and effect are significant different (P < 0.05).
